# Peripheral Venous Catheter-Related Adverse Events: Evaluation from a Multicentre Epidemiological Study in France (the CATHEVAL Project)

**DOI:** 10.1371/journal.pone.0168637

**Published:** 2017-01-03

**Authors:** Katiuska Miliani, Raphaël Taravella, Denis Thillard, Valérie Chauvin, Emmanuelle Martin, Stéphanie Edouard, Pascal Astagneau, on behalf of the CATHEVAL Study Group

**Affiliations:** 1 Coordinating Centre for control of healthcare-associated infections (CClin), Assistance Publique-Hôpitaux de Paris (AP-HP), Paris, France; 2 Infection Control Department, Lariboisière/F. Widal University Teaching Hospital, Paris, France; 3 Infection Control Department, Elbeuf-Louviers-Val de Reuil Intercommunal Hospital, Elbeuf, France; 4 Infection Control Department, Dieppe Hospital, Dieppe, France; Nanjing University Medical School Affiliated Nanjing Drum Tower Hospital, CHINA

## Abstract

**Introduction:**

Peripheral venous catheters (PVC) are medical devices most frequently used during hospital care. Although the frequency of specific PVC-related adverse events (PVCAEs) has been reported, the global risk related to the insertion of this device is poorly estimated. The aim of this study is to determine the incidence of PVCAEs during the indwell time, after catheter removal, and to identify practice-mirroring risk factors.

**Methods:**

A prospective observational study was conducted as a part of a research project, called CATHEVAL, in one surgery ward and four medicine wards from three public general tertiary care hospitals in Northern France that were invited to participate between June-2013 and June-2014. Each participating ward included during a two-month study period all patients older than 15 years carrying a PVC. All inserted PVCs were monitored from insertion of PVC to up to 48 hours after removal. Monitored data included several practice-mirroring items, as well as the occurrence of at least one PVCAE. A multivariate Cox proportional hazard model, based on a marginal risk approach, was used to identify factors associated with the occurrence of at least one PVCAE.

**Results:**

Data were analysed for 815 PVCs (1964 PVC-days) in 573 patients. The incidence of PVCAE was 52.3/100 PVCs (21.9/100 PVC-days). PVCAEs were mainly clinical: phlebitis (20.1/100 PVCs), haematoma (17.7/100 PVCs) and liquid/blood escape (13.1/100 PVCs). Infections accounted for only 0.4/100 PVCs. The most frequent mechanical PVCAEs, was obstruction/occlusion of PVC (12.4/100 PVCs). The incidence of post-removal PVCAEs was 21.7/100 PVCs. Unstable PVC and unclean dressing were the two main risk factors.

**Conclusion:**

Limitation of breaches in healthcare quality including post-removal monitoring should be reinforced to prevent PVC-related adverse events in hospital settings.

## Introduction

Peripheral venous catheters (PVC) are the most frequently used medical devices during hospital cares [1]. In France, up to 25 million of PVCs are placed every year [2] and nearly 20% of hospitalized patients carry such a device [3]. Although such devices are frequently used and often considered as presenting a low risk to the patient, PVCs are associated with significant adverse events [4,5] that may impair treatment administration and patient health. A wide range of PVC-related adverse events (PVCAEs) has been reported including partial dislodgement or accidental removal, phlebitis (irritation or inflammation to the vein wall), occlusion (blockage), infiltration (fluid moving into surrounding tissue), fluid or blood leakage and, rarely, infections [4,6,7]. So far, most studies have been focused on specific events, such as phlebitis [4,5,8–10], infiltration [5,8–10] and PVC-related infections [5,8,11–13].

According to some studies, phlebitis incidence rates varied widely from 2% to 80%, depending on the definitions used [14]. Similarly, incidence rates of infiltration reach up to 30% of inserted PVCs [4,9]. In contrast, peripheral lines are less frequently related to infection as compared to central line, but serious sepsis may occur in some cases [11]. Whatever the study design and population, the overall rate of PVCAEs has not been clearly estimated as a part of a composite measure of clinical and mechanical incidents including events after PVC withdrawal.

In France, a nationwide audit of PVC insertion and maintenance practices in healthcare settings was endorsed by the national program for prevention of healthcare-associated infection [15]. Following this initiative, a research project called CATHEVAL was launched in a subset of voluntary hospitals in order to determine the relationship between PVC-related practices and occurrence of adverse events during and after device insertion. As a part of a multidimensional project including quantitative and qualitative approach, we present here the epidemiological aspects of the study including incidence and practice-mirroring risk factors.

## Materials and Methods

### Aim

The aim of this study is to determine the incidence of PVCAEs during the indwell time and after catheter removal and to identify practice-mirroring risk factors.

### Study design and participants

A prospective observational study was conducted in one surgery ward and four medicine wards from three public general tertiary care hospitals in Northern France that were invited to participate between June 2013 and June 2014. Each participating ward included during a 2-month study period all patients older than 15 years who were hospitalized in the ward while carrying a PVC. Every PVC of each patient was included as long as indwell was expected to exceed 24 hours. Members of Infection control team (ICT) collected all data after informing the patient or their legal guardian about the study and obtaining oral consent. They also carried out the daily monitoring of all inserted PVC. Data were collected on standardized questionnaires which were filled out on the basis of information obtained from discussions with patients and paramedical staff taking care of them and by inspecting the catheter insertion sites. The members of ICT were not involved in the patient medical care or in the decision to remove intravenous catheters.

### Ethics statement

This study was not subject to the approval of an ethic committee. In accordance with French legislation for biomedical research and human subject research at the starting time of the study, non-interventional studies (observational), in contrast with interventional ones, were exempt from this mandatory requirement.

The study obtained both an agreement from the French Advisory Committee for Data Processing in Health Research (CCTIRS, Paris, September 2013, Agreement No. 13.719) and clearance from the French Data Protection Authority (CNIL, Paris, October 2014, Authorisation No.914068. The request for the authorization was submitted on January 2014 and registered under the registration No. rei0882801y), as required for protection of personal data.

All patients were informed about the purpose of the study through an information letter. For minors, two information letters were available: one for the young and the other for their parents or their legal guardian. Oral consent was obtained from patients or their legal guardian (in the case of minors or for patients who were unable to consent themselves) before study participation. Patients who accepted to participate in the study were progressively included as they arrived in the hospital ward during the two-month study period. Patients who refused to participate in the study, those younger than 15 years and those for which verbal consent was not obtained from their guardian (in the case of minors or for patients who were unable to consent themselves) were not included.

As the members of ICT were not involved in the patient medical care, their only interactions with patients were those related to inspecting catheter insertion sites, in order to assess practice compliance with national guidelines for the prevention of PVC-related infections [2], and to collecting patient symptoms, including pain.

The "CATHEVAL" research project was publicly funded by a grant from the Research Program on the Evaluation of the Healthcare System Performance, Ministry of Health, France (grant No. PREPS-12-002-0037). Assistance Publique—Hôpitaux de Paris (Département de la Recherche Clinique et du Développement) sponsored the project. The funders had no role in the study design, data collection and analysis, decision to publish, or preparation of the manuscript.

### Data collection

Data collected included individual patient and PVC items. Patients data were date of hospital admission and discharge, date of inclusion in the study, age, gender, skin conditions, venous capital, Charlson comorbidity score [16], previous PVC during the current hospitalization, and whether the patient had current infections or a behavioural disorder. PVC items were dates of insertion and removal, insertion site, PVC rank, PVC monitoring data and reasons for catheter withdrawal. PVC monitoring data included several practice-mirroring items and the occurrence of mechanical or clinical PVCAEs. The latter were first recorded as suspected events based on daily collection of the presence/absence of signs and/or symptoms during indwell time to up to 48 hours after catheter removal. The following signs and symptoms could be recorded: redness/warmth, tenderness/pain, œdema/swelling, induration, palpable venous cord, presence of pus, clearer skin, fluid/blood leaking, haematoma, and fever (when concomitant with another symptom or sign). Clinical PVCAEs were finally considered as isolated oedema, phlebitis, haematoma at the insertion site, fluid/blood leaking at insertion site and suspected sepsis. Compared to collection of monitoring data of clinical PVCAEs, collection of monitoring data of mechanical PVCAES as well as of practice-mirroring items could occur during indwell time only. The following mechanical events could be reported: accidental/wrenching removal, obstruction/occlusion, blood reflux, rupture of closed circuit system or other mechanical events (*e*.*g*. catheter rupture). In regard to the collected practice-mirroring items, these were related to: i) PVC fixation (whether stable/fixed); ii) dressing regimes of insertion site (whether clean, or occlusive, or transparent dressing); iii) condition/position of infusion devices (whether infusion stand, or protective casing, or maintained closed circuit system, and whether infusion lines, stopcocks are lying in the bed or close to an infectiously hazardous area).

### Outcomes

The main outcome was the occurrence of at least one PVCAE during catheter indwell or within 48 hours after removal. PVCAEs could be clinical or mechanical. The assessed clinical PVCAE were isolated oedema, phlebitis, haematoma at the insertion site, fluid/blood leaking at insertion site, and suspected sepsis. An oedema at the insertion site was defined as the swelling of the tissue around the PVC insertion site usually as a result of intravenous fluids leaking into the tissues. It was considered as a fully-fledged PVCAE when it was observed in the absence of any other signs. Phlebitis, which is the irritation and inflammation of a vein wall caused by the presence of the PVC [1,7], was assessed based on Ray Maddox phlebitis grading scale [17] with minor modifications (see Table 1). The scale graded phlebitis according to five levels of severity based on the presence or absence of the following symptoms: pain at venepuncture site, erythema, swelling, induration, palpable venous cord, pus at the insertion site. According to the level allocated to phlebitis, two groups were distinguished: suspected phlebitis for those showing a grade 1 or 2 and manifest phlebitis for those graded 3 or greater. A haematoma at the insertion site was defined as a localized collection of extravasated blood around the PVC insertion site resulting from a leakage of blood from the blood vessel into the surrounding soft tissue. Fluid/blood leaking at insertion site was defined as the inadvertent leakage from the insertion site of intravenous fluids or blood visible through the dressing. Finally, infectious PCAES were defined based on clinical criteria only and considered as suspected events because PVC-tip cultures are not routinely performed in the participating hospitals Thus, local infections at the insertion site were accounted as a grade 5 phlebitis (i.e. pus at the insertion site with all sign/symptoms of grade 4 phlebitis: pain at the insertion site with erythema and swelling and induration and palpable venous cord) and when a grade 2 or greater phlebitis (see Table 1) was present with a concomitant fever, it was considered as a suspected sepsis.

**Table 1:** Criteria for judging phlebitis based on Ray R. Maddox phlebitis grading scale [17] with minor modifications*.

Grade	Description
**1**	Painful insertion site, no erythema, no swelling, no induration, no palpable venous cord
**2**	Painful insertion site with erythema or some degree of swelling or both, no induration, no palpable venous cord
**3**	Painful insertion site with erythema and swelling and induration and no palpable venous cord
**4**	Painful insertion site with erythema and swelling and induration and palpable venous cord
**5**	Pus at the insertion site with all sign/symptoms of grade 4

* The levels 3 to 5 of the Maddox scale [17] were slightly simplified in our study as the size of palpable venous cords was not collected, and the pus at the insertion site was added as a sign of gravity in the grade 5.

Regarding mechanical PVCAEs, it was defined as an accidental/wrenching removal, all catheter dislodgement that was not planned; an obstruction/occlusion of PVC as the inability to infuse intravenous fluids leading to PVC withdrawal and a rupture of closed circuit system when infusion lines were detached or disconnected from the infusion bag or in the absence of a well-sealed injection site.

Secondary endpoints were the practice-mirroring data, which were used as potential predictor variables of PVCAEs related to a breach in PVC upkeep quality according to the joint recommendations of the French society for hospital hygiene and the national authority of health [2]. A quality breach was defined as: i) at least one negative response to the following items: stabilized PVC, clean dressing, occlusive dressing, transparent dressing, infusion stand, protective casing, maintained closed circuit system; ii) at least one positive response to the following items: infusion lines, stopcocks are lying in the bed; infusion lines, stopcocks are close to an infectious hazardous area. The blood reflux, initially collected as a mechanical adverse event, was instead considered as a breach in the quality of care if observed at least once during the indwell time.

### Data analysis

Statistical analysis was performed using STATA/IC 11.2 StataCorp LP, College Station, USA. All variables were considered as categorical. Continuous variables were transformed into categorical variables by classing the variable or by splitting them according to the median or the quartiles of their distribution. PVC items were analysed on the condition that PVC had been monitored at least once during indwell. All practice-mirroring items were transformed into dichotomous variables and considered present if occurring at least once. The incidence rate per 100 PVC, and the incidence density per 100 PVC-days, and the binomial exact 95% confidence interval (95% CI) were calculated. PVCAEs occurring only within 48 hours after removal were analysed separately in PVCs meeting the following criteria: no occurrence of PVCAEs during the indwell time and complete recording of daily PVC monitoring data during the indwell time. Kaplan-Meier curves and Log-Rank test were assessed to compare time until first PVCAE occurrence per event class. Thus, the study population was split into three groups: PVCs with only mechanical PVCAEs, PVCs with only clinical PVCAEs, and PVCs with both mechanical and clinical PVCAEs. When more than one event occurred, the date of first detection was only considered. Univariate analysis was conducted using Pearson’s Chi-square test or Fisher’s exact test. Multivariate analysis was performed using a multivariate Cox proportional hazards model with marginal risk set to take into account multiple correlated failure-time data using the Wei, Lin & Weissfeld approach [18]. The id-patient variable was used to cluster the related observations when estimating the Cox model. The initial model considered all significant variables at level *p<*0.25. Cox regression hypotheses were validated through a proportional hazard assumption test and graphical analysis of Schoenfeld residuals. Model fitness was assessed using a specification test and graphical analyses of Cox-Snell residuals. All final results were considered significant when *p*<0.05.

## Results

Data were collected from 856 PVCs inserted in 586 patients. After exclusion of 41 PVCs (13 patients) because of missing data at insertion or during the indwell time, 815 PVCs (1964 PVC-days) in 573 patients were finally analysed. Most study patients (98%) had only one hospital stay during the study period, 33% had a limited venous capital, 23% a current infection, 19% cutaneous lesions (mainly haematomas), 6% presented a behavioural disorder, and 54% were male. Half of patients had a Charlson comorbidity score between 0 and 1, 32% between 2 and 3, and 18% more than 3 (Table 2). The median patient age was 69 years (interquartile range [IQR]: 56–81, range 16–100). Overall, the median number of catheters per patient was 1 (IQR: 1–2; range: 1–7).

**Table 2:** Demographics and clinical characteristics of patients, N = 573 patients.

Variables	
**Number of stays, *n (%)***	
*** 1***	562 (98.1)
*** 2***	9 (1.6)
*** 3***	2 (0.4)
**Age (years)**	
*** Mean (SD)***	67.0 (17.1)
*** Median (IQR)***	69 (56–81)
**Gender, *n (%)***	
*** Female***	264 (46.1)
*** Male***	309 (53.9)
**Charlson comorbidity score, *n (%)***	
*** 0***	144 (25.1)
*** 1***	140 (24.4)
*** 2***	122 (21.3)
*** 3***	61 (10.7)
*** 4 and more***	106 (18.5)
**Venous capital, *n (%)***	
*** Normal***	380 (66.3)
*** Limited***	188 (32.8)
*** Missing***	5 (0.9)
**Skin condition, *n (%)***	
*** Healthy***	455 (79.4)
*** Lesions***	13 (2.3)
*** Haematomas***	92 (16.1)
*** Not recorded***	13 (2.3)
**Current infection, *n (%)***	
*** No***	433 (75.6)
*** Yes***	128 (22.3)
*** Missing***	12 (2.1)
**Transmission-based precautions, *n (%)***	40 (7)
*** Contact***	23 (4)
*** Droplet***	0
*** Airborne***	3 (0.5)
*** Protective confinement***	0
*** Not specified***	17 (3)
**Behavioural disorder, *n (%)***	
*** No***	534 (93.2)
*** Yes***	35 (6.1)
*** Missing***	4 (0.7)
**Number of inserted PVCs, *n (%)***	
*** 1***	393 (68.6)
*** 2***	137 (23.9)
*** 3 and more***	43 (7.5)

**SD:** Standard deviation; **IQR:** interquartile range

Three quarters of PVCs (77%) were placed in the 3 medical wards and 23% in the surgical. More than a half of PVCs were inserted on the forearm veins followed by the hand dorsum, the wrist, and the antecubital fossa. The median dwell time was two days (IQR: 1–3), however, a dwell time of more than 4 days was observed in 77 (9.5%) of PVCs, of which 61% had been inserted in patients aged more than 69 years. A total of 753 (92.4%) PVCs were exposed to at least one quality breach including 171 (23%) exposed to one, 279 (37%) to two, and 303 (40%) to more than two. The more frequent quality breaches were related to condition/position of infusion devices (tubing related) and/or dressing regimens (dressing related) (Table 3). For a total of 721 (88.5%) PVCs, the reason for withdrawal was obtained. The most frequently reported reason was the end of intravenous treatment or unnecessary PVC (64.9%) followed by infusion failure because of occurrence of a clinical or mechanical PVCAE (27.2%) and preventive withdrawal (PVC dwelling ≥ 96 hours) (5%). Post-removal surveillance was performed in 62.5% of PVCs monitored.

**Table 3:** Frequency of quality breaches observed at least once during dwelling time in 815 PVCs.

	No.	%	95% CI
**Catheter related**			
*** *-Unstable PVC**	35	4.3	[3.0–5.9]
**Dressing related**			
*** *-Non-occlusive dressing**	163	20.0	[17.3–22.9]
*** *-Unclean dressing**	149	18.3	[15.7–21.1]
*** *-Covered transparent dressing** ^**a**^	30	3.7	[2.5–5.2]
**Tubing related**			
*** *-Absent infusion stand**	689	84.5	[81.9–87.0]
*** *-Infusion lines, stopcocks were lying in the bed without protective casing**	491	60.2	[56.8–63.6]
*** *-Closed circuit system rupture** ^**b**^	25	3.1	[2.0–4.5]
*** *-Infusion lines, stopcocks were close to an infectious hazardous area**	17	2.1	[1.2–3.3]
**Care related**			
*** *-Blood reflux**	218	26.7	[23.7–29.9]

^a^ This is a transparent dressing which was covered during indwell by a bandage placed thereon.

^b^ When the closed circuit system is broken, for example, by the use of a mechanical device like an infusion pump.

**95% CI:** Confidence interval; **PVC:** Peripheral venous catheter.

**Fig 1:**
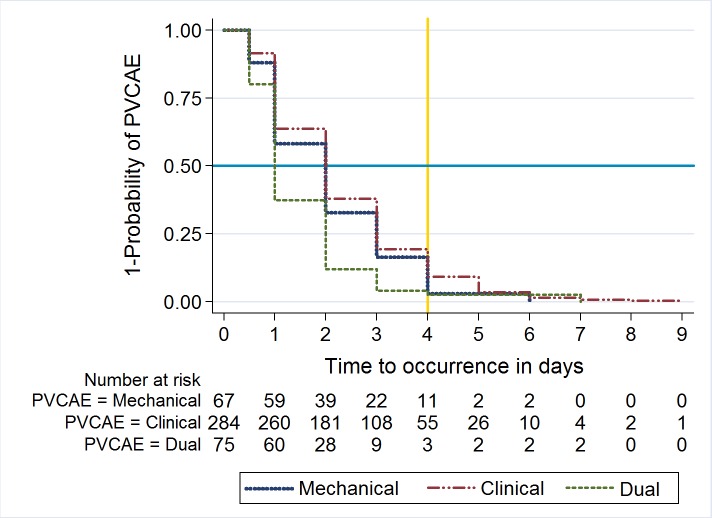
Kaplan-Meier estimates of time to first PVC-related adverse event occurrence per event class. **Legend**: Kaplan-Meier estimates took into account only PVCs with at least one adverse event in order to compare time according event class, including those occurred during post-removal follow-up. The blue line represents the 50% mark and the yellow line the recommended delay for routine removal of PVCs. PVC: Peripheral venous catheter; PVCAEs: PVC-related adverse events. **Note:** Mechanical PVCAEs could occur only during indwell only.

An average of 1.4 PVCAEs per PVC were observed. The incidence rate of at least one PVCAE was 52.3 per 100 PVCS; 95% CI: 48.8–55.7 (Table 4). Clinical PVCAEs were significantly more frequent than mechanical ones (*p*<0.001). The most frequent clinical PVCAEs were phlebitis, followed by haematoma and fluid/blood leaking whereas obstruction/occlusion of PVC was the most frequent mechanical PVCAEs. According to the five levels of phlebitis grading scale, there were 35 (21.3%) graded 1; 95 (57.9%) graded 2; 19 (11.6%) graded 3; 14 (8.6%) graded 4; 1 (0.6%) graded 5. Suspected phlebitis cases (grade 1 or 2) were more frequent than manifest phlebitis (grade 3 or greater) (Table 4). Most PVCAEs occurred before the second day after PVC insertion (Fig 1). No significant difference was observed between time occurrence of clinical and mechanical PVCAEs (*Log-Rank test p = 0*.*20)*. However, dual PVCAEs occurred significantly earlier than single PVCAEs *i*.*e*. clinical or mechanical (*Log-Rank test p*<0.001).

**Table 4:** Incidence of PVCAEs according to class and type occurring during entire follow-up ^a^, N = 815 PVCs (1964 PVC-days).

	No. PVCAEs	Incidence per 100 PVCs	95% CI	Incidence per 100 PVC-days	95% CI
**PVCAEs class (at least one)**					
* ***Any**	**426**	**52.3**	**[48.8; 55.7]**	**21.7**	**[19.9–23.6]**
* ***Mechanical**	67	8.2	[6.4; 10.3]	3.4	[2.7–4.3]
* ***Clinical**	284	34.8	[31.6; 38.2]	14.5	[12.9–16.1]
* ***Dual**	75	9.2	[7.3; 11.4]	3.8	[3.0–4.8]
**PVCAEs type**					
* ***Isolated oedema**	31	3.8	[2.6; 5.4]	1.6	[1.1–2.2]
* ***Phlebitis**	164	20.1	[17.4; 23.0]	8.4	[7.2–9.7]
* ****Suspected phlebitis*** ^b^	*130*	*16*.*0*	*[13*.*5; 18*.*6]*	*6*.*6*	*[5*.*6–7*.*8]*
* ****Manifest phlebitis*** ^c^	*34*	*4*.*1*	*[2*.*9; 5*.*8]*	*1*.*7*	*[1*.*2–2*.*4]*
* ***Haematoma**	144	17.7	[15.1; 20.5]	7.3	[6.2–8.6]
* ***Suspected sepsis**	3	0.4	[0.1; 1.1]	0.2	[0.03–0.4]
* ***Fluid/Blood leaking**	107	13.1	[10.9; 15.6]	5.4	[4.4–6.5]
* ***Accidental/wrenching removal**	26	3.2	[2.1; 4.6]	1.3	[0.9–1.9]
* ***Obstruction/occlusion**	101	12.4	[10.2; 14.9]	5.1	[4.2–6.2]
* ***Closed system rupture and other mechanical incidents**	22	2.7	[1.7; 4.1]	1.1	[0.7–1.7]

^a^ From PVC insertion to up to 48 hours after withdrawal

^b^ Suspected phlebitis: those showing a grade 1 or a grade 2 according to phlebitis grading scale (see Table 1).

^c^ Manifest phlebitis: those showing a grade >2 according to phlebitis grading scale (see Table 1).

**PVCAEs:** Peripheral venous catheter adverse events.

**95% CI:** 95% confidence interval.

Univariate analysis comparing incidence of PVCAEs according to patient characteristics showed that PVCAE incidence was higher in female and older patients (>69 years of age), with limited venous capital, having cutaneous lesions or haematomas and in patients with behavioural disorders. The PVCAE incidence was not significantly different between participating hospitals or wards according to number of stays, the Charlson comorbidity score or the anatomical insertion site of PVCs (Table 5). Univariate analysis comparing incidence of PVCAEs according to practice-mirroring variables showed that PVCAE incidence was also higher in patients having unstable PVCs, non-occlusive or unclean dressings, and with a dwell time more than two days whereas patients having infusion lines and stopcocks lying in the bed without protective casing were associated with a lower PVCAE incidence (Table 6).

**Table 5:** Incidence of at least one PVCAE according to patient characteristics in univariate analysis, n = 815 PVCs.

Variables	All PVCs (n = 815)	PVCAEs per 100 PVCs ^a^	*P* ^b^	Variables	All PVCs (n = 815)	PVCAEs per 100 PVCs ^a^	*P* ^b^
**Hospital**				**Venous capital**			
* ***A**	381	49.3	0.26	** Normal**	525	46.3	**<0.001**
* ***B**	314	54.1		** Limited**	282	63.5	
* ***C**	120	56.7		** Missing**	8	50.0	
**Location (ward) in hospital**				**Skin condition**			
* ***Surgery (hospital A)**	187	51.3	0.35	** Healthy**	612	47.6	**<0.001**
* ***Medicine (hospital A)**	194	47.4		** Cutaneous lesions**	24	66.7	
* ***Cardiology d (hospital B)**	314	54.1		** Haematomas**	158	67.1	
* ***Cardiology (hospital C)**	120	56.7		** Missing**	21	61.9	
**Number of stays**				**Current infection**			
* ***1**	784	52.6	0.69	** No**	601	51.6	0.95
* ***2**	23	43.5		** Yes**	193	51.3	
* ***3**	8	50.0		** Missing**	21	81.0	
**Age (years)**				**Behavioural disorder**			
**≤56**	187	42.8	**0.001**	** No**	739	50.7	**<0.01**
* ***57–69**	206	47.6		** Yes**	69	68.1	
* ***70–81**	218	55.5		** Missing**	7	57.1	
* ***>81**	204	62.3		**Previous PVC**			
**Gender**				** No**	392	49.2	0.12
* ***Male**	448	48.4	**0.02**	** Yes**	413	54.7	
* ***Female**	367	57.0		** Missing**	10	70.0	
**Charlson comorbidity score**				**Anatomical insertion site**			
* ***0**	177	49.7	0.51	** Forearm**	449	51.0	0.69
* ***1**	203	50.7		** Dorsum of the hand**	145	55.2	
* ***2**	176	54.0		** Antecubital fossa**	95	54.7	
* ***3**	88	47.7		** Wrist**	118	50.0	
* ***4 or more**	171	57.3		** Other sites**	5	80.0	
				** Missing**	3	66.7	

^a^ Incidence of at least one PVCAE during entire follow-up (from PVC insertion until 48 hours after withdrawal).

^b^ Pearson’s Chi-square test.

**PVC:** Peripheral venous catheters; **PVCAEs:** Peripheral venous catheter-related adverse events.

**Table 6:** Incidence of at least one PVCAE according to practice-mirroring variables in univariate analysis, n = 815 PVCs.

Variables	All PVCs (n = 815)	PVCAEs per 100 PVCs ^a^	*P* ^b^	Variables	All PVCs (n = 815)	PVCAEs per 100 PVCs ^a^	*P* ^b^
**Unstable PVC**				**Closed system rupture**			
* ***No**	780	51.2	**<0.01**	** No**	790	51.9	0.23
* ***Yes**	35	77.1		** Yes**	25	64	
**Non-occlusive dressing**				**Infusion lines, stopcocks were close to an infectiously hazardous area**			
* ***No**	652	50.3	**0.03**	** No**	798	52.5	0.36
* ***Yes**	163	60.1		** Yes**	17	41.2	
**Unclean dressing**				**Blood reflux**			
* ***No**	666	47.5	**<0.001**	** No**	586	51.9	0.99
* ***Yes**	149	73.8		** Yes**	218	51.8	
**Covered transparent dressing** ^c^				** Missing**	11	81.8	
* ***No**	785	51.9	0.22	**Indwelling time**			
* ***Yes**	30	63.3		** ≤2 days**	502	47.4	<0.01
**Absent infusion stand**				** 3 days**	147	61.9	
* ***No**	126	59.5	0.08	** 4 days**	89	59.6	
* ***Yes**	689	50.9		** 5 or more days**	77	57.1	
**Infusion lines, stopcocks were lying in the bed without protective casing**				**Order of worn PVC**			
				** 1**^**st**^	566	52.5	0.33
* ***No**	324	59.6	**<0.001**	** 2**^**nd**^	178	48.9	
* ***Yes**	491	47.5		** 3**^**rd**^ **and subsequent**	71	59.2	

^a^ Incidence of at least one PVCAE during entire follow-up (from PVC insertion until 48 hours after withdrawal).

^b^ Pearson’s Chi-square test.

^c^ This is a transparent dressing which was covered during indwell by a bandage placed thereon.

**PVC:** Peripheral venous catheters; **PVCAEs:** Peripheral venous catheter-related adverse events

In the multivariate analysis, age was found as a significant statistical interaction. Therefore, the analysis was performed in two separate groups according to an age cut-off of 70 years (Table 7). Overall, 760 PVCs were analysed with no missing data, including 377 and 383 PVCs respectively within the two age groups. Overall, nine risk factors were kept in the final models. Among patients less than 70 years, being female, having limited venous capital, unclean dressing, and unstable catheter were independent risk factors associated with higher risk whereas a covered transparent dressing was protective factor of PVCAEs. Among aged 70 years or over patients, cutaneous lesions, PVC insertion at the antecubital fossa, and an unclean dressing were independent risk factors of PVCAE. The insertion of PVC at the dorsum of the hand or at the wrist were not significantly associated with PVCAEs (*p* = 0.10 and *p* = 0.94, respectively). Infusion lines, stopcocks lying in the bed without protective casings, and an indwell time greater than four days were independently associated with a reduced risk of PVCAE in both age groups.

**Table 7:** Risk factors of PVCAEs according to age groups ^a^, in multivariate analysis.

Risk factor	Patients <70 years ^b^	*p*	Patients ≥ 70 years ^b^	*p*
	Adjusted HR [95% CI]		Adjusted HR [95% CI]	
**Female**	1.87 (1.32–2.64)	<0.001	(-)	
**Limited venous capital**	1.68 (1.21–2.34)	0.002	(-)	
**Indwell time greater than 4 days**	0.26 (0.14–0.48)	<0.001	0.32 (0.19–0.53)	<0.001
**Cutaneous lesions**	(-)		1.41 (1.04–1.91)	0.03
**Insertion site**				
* ***Forearm**	(-)		Reference	0.03
* ***Dorsum of the hand**	(-)		1.39 (0.94–2.04)	
* ***Antecubital fossa / other sites**	(-)		1.72 (1.14–2.59)	
* ***Wrist**	(-)		0.98 (0.64–1.52)	
**Unstable catheter**	4.93 (3.13–7.77)	<0.001	(-)	
**Unclean dressing**	2.13 (1.47–3.10)	<0.001	1.66 (1.23–2.24)	0.001
**Covered transparent dressing** ^c^	0.06 (0.02–0.19)	<0.001	(-)	
**Infusion lines, stopcock lying in the bed without protective casing**	0.59 (0.42–0.82)	0.002	0.63 (0.47–0.86)	<0.01
**Model validation results**				
**Number of PVCs**	377		383	
**Number of patients**	280		259	
**Number of PVCs with at least one PVCAE**	171		225	
**Likelihood-ratio test (full *vs* final model)**	Chi2 = 5.28 (9 df), *p* = 0.81		Chi2 = 2.88 (9 df), *p* = 0.97	
**Akaike information criterion (full *vs* final model)**	1404.08; 1391.35		1923.64; 1908.52	
**Global test of proportional-hazards assumption**	Chi2 = 6.56 (7 df), *p* = 0.47		Chi2 = 25.31 (7 df), *p* = 0.62	

**Note:** Findings are from a multivariate Cox proportional hazards regression model with marginal risk sets using the Wei, Lin & Weissfeld approach [18].

^a^ In the first multivariate models, age was found as a significant statistical interaction. Therefore, the analysis was performed in two separate groups according to an age cut-off of 70 years (median patient age)

^b^ Final model. Full model included all variables at *p*≤0.25 in univariate analysis (except age) and the anatomical insertion site.

^c^ This is a transparent dressing which was covered during indwell by a bandage placed thereon.

**95% CI:** Confidence interval; **df:** degrees of freedom; **HR:** hazard ratio; **PVC:** Peripheral venous catheters; **PVCAEs:** Peripheral venous catheter-related adverse events

Incidence of post-removal PVCAE only was estimated in a subset of 359 PVCs that met the selection criteria. In this sample, the incidence rate was 21.7 per 100 PVCs (95% CI: 17.6–26.4). The most frequent clinical PVCAEs were haematoma at the insertion site and phlebitis (incidence rates: 12.5 (95% CI: 9.3–16.4) and 9.5 per 100 PVCs (95% CI: 6.6–13.0), respectively). According to the level of severity, phlebitis cases (n = 34) were distributed as follows: 12% grade 1; 65% grade 2; 17% grade 3 and 6% grade 4.

## Discussion

We reported here a three-times greater incidence of PVCAEs than those reported in the few studies already published using a composite measure of clinical and mechanical adverse events [4,6,9]. This apparent discrepancy could be explained by different reasons. First, PVCAEs definition included a very large set of adverse events including mechanical and clinical events, especially haematoma and early level of phlebitis which could enhance the incidence rate, as compared to other studies. Second, our study was based on a prospective survey of PVCs with an active monitoring of clinical signs defining adverse events from insertion up to 48 after removal and few data were missing. Third, post-removal PVCAEs were also traced, resulting in almost a 20% gain in incidence.

In our study, the most frequent PVCAE was a phlebitis which occurred in one out of five PVCs. This result is consistent with that reported in other studies [4,8,9,14,19], even though comparisons should be made with caution because of the heterogeneity of definition criteria [14,20]. In contrast to other studies, we adopted a relatively large and sensitive case definition including pain at the venepuncture site without other symptoms considered as first-level phlebitis [8,21,22]. Indeed, we considered early clinical symptoms of phlebitis as an adverse event related to PVC because, in 42% of « suspected phlebitis » (grade 1 or 2 of our adapted Maddox phlebitis scale), the device was withdrawn before that a more severe phlebitis occurred. In the study by Uslusoy *et al* [23], the scale of phlebitis according to Intravenous Nurse Society criteria was based on wider definition including redness and/or pain on the catheterization site at the first degree. Then phlebitis rate per 100 PVCs was twice higher than our rate including half of the first degree. Conversely, only 4% developed phlebitis after PVC removal [23] which is lower than those found in our study. Another study by Hershey *et al* [22] reported that more than 40% of phlebitis cases occurred more than 24 hours after PVC withdrawal whereas in the study by Webster *et al* [24], 75% of cases of phlebitis were diagnosed in individuals who did not have phlebitis when the catheter was removed. However, their rate of phlebitis at 48 hours (1.8%) was lower than our rate of 20.1 per 100 PVCs.

The second most common PVCAE was the occurrence of haematoma at the insertion site. Among studies which considered haematoma as a complication related to PVC insertion site [25,26], one study reported [26] that patients who received anticoagulation therapy have developed haematoma at the hand dorsum that required surgical evacuation. Although some patient conditions can predispose to the development of haematoma (*e*.*g*. coagulation profile in excess of the therapeutic range, or if patient received anticoagulation therapy), haematomas could be indicative of the quality of catheter handling during insertion and removal with potential serious consequences including skin necrosis [26]. In our study, nearly a third of haematomas occurred after PVC removal suggesting an inadequate venous compression at the removal site. However, half of PVCs were assessed in cardiology wards, where patients usually receive anticoagulation therapy. For these patients, nurses and patients should be particularly aware of doing prompt and prolonged compression at the insertion site after PVC removal, especially in older patients with impaired skin conditions.

Infectious PVCAEs were one of the least frequent events in our study. Although definition of infectious PVCAEs were based on clinical signs, incidence rate of local infection (grade 5 phlebitis) or systemic infection (suspicion of sepsis) was consistent with many other studies [5,8,11–13] where infection rates (including PVC-related bloodstream infection) were estimated to be 0.1 per 100 PVCs and 0.5 per 1000 PVC-days [11]. However, severe sepsis is a critical event which could be associated with significant mortality. Given that millions of PVCs are inserted every year, the absolute number of PVC-related severe infectious events could become of immediate concern in terms of human and economic burden. Based on a case-fatality rate associated with bloodstream infection of 15% [27], 150 deaths per million PVCs would occur each year, which would result in more than 3 000 deaths in France.

Two main risk factors of PVCAE were related to failure in device handling reflecting breaches in the upkeep quality. The first was the unstable insertion of PVC within a vein which is a potential cause of damage of the vessel wall. The release of thromboplastic substances and platelets promotes blood clotting and may cause the constriction and occlusion of the catheterized vein. This may result in the leakage of intravenous fluids from the insertion site, or their infiltration into the surrounding tissues, and subsequently impairment of venous access, as reported in our study and described in others [1,7]. The second was the soiled/unclean dressing observed during the indwell time. This situation could provide an ideal opportunity for increasing infection-producing microorganisms [1,7], especially when non-transparent dressings are used, increasing the risk of not detecting infection. In France as in other countries, guidelines recommend the use of transparent, semi-permeable polyurethane dressings [2]. In our study, 3.7% of PVCs were covered by a bandage placed on the transparent dressing which was used when inserting the PVC for any medical reasons. This uncommon practice was associated with a reduced risk of PVCAEs in patients less than 70 years. Whether the type of dressings or new generation of safety devices is better than any other in securing PVCs would require further discussion, as pointed out in a recent review [7].

The insertion site at the antecubital fossa was found to be a risk factor associated with higher risk of PVCAEs in the older patients. This result remains controversial in many studies. For phlebitis, some studies found that insertion at forearm and antecubital fossa was associated with a higher risk compared with hand dorsum or wrist [6,23], whereas insertion at the hand dorsum and the antecubital fossa was associated with occlusion and accidental removal but not with phlebitis [21]. Another study [28] reported that PVC inserted at the antecubital fossa and the forearm veins is associated with a lower risk of phlebitis compared with the hand dorsum. According to our results, we would recommend to use whenever possible to sites other than antecubital fossa, especially in older patients.

Surprisingly, the infusion lines and stopcocks lying in the bed without protective casing were associated with a reduced risk of PVCAE. To our knowledge, this factor has never been reported to be associated with clinical or mechanical risk in other studies. Although this apparently paradoxical situation may increase the risk of sepsis, we assume that the infusion lines were placed far from infectious sites such as wound or ostomy. For any reasons, the patients with such material should be under closely PVC surveillance at the bedside by the clinical staff because the infusion lines were without protection.

Although the relationship between infection risk and indwell time has been evidenced with central venous catheters, the risk associated with PVC is not so clearly established. In our study, we found that indwell time greater than four days was associated with a lower risk of PVCAE. The fact that most PVCAEs occurred within the first four days after catheter insertion could partly explain this negative association, since half PVCs are removed after two days. Recent studies [5,10] showed no benefit of routine replacement (i.e. between 72–96 hours) [29] and have suggested that clinically indicated replacement is safe and would spare patients the unnecessary pain of routine re-sites in the absence of clinical indications which would also provide significant cost savings.

There are some limitations to our study. Firstly, our study did not take into account some previously identified risk factors, such as type of infusate, catheter gauge, catheter material, type of cannula dressing, or care provider [6,21,23,28]. However, our purpose was not to study all known factors, but to concentrate on the possible association between PVCAE occurrence and some practice-mirroring factors, which have been poorly analysed until now. Secondly, PVCAE definitions were mainly based on clinical criteria. Sepsis was defined only according to signs at the insertion site associated (grade 2 or greater phlebitis) with fever without any microbiological criteria because PVC-tip cultures were not routinely performed in the participating hospitals. In addition, we have not collected the way the pain at the insertion site was assessed, the measurement of the size of redness, swelling nor length of palpable venous cordon, which are usually considered when evaluating phlebitis. However, our definition of phlebitis was based on a standard scale, grading the severity of clinical signs.

## Conclusion

Despite some limitations due to non-experimental study design with clinical-based definition criteria, our study suggested that the incidence of PVC-related adverse events are currently underestimated in healthcare settings. PVCAEs in our hospital population were frequent, although infectious events remain rare. Furthermore, the practice-mirroring risk factors determined in this study demonstrate the importance of quality PVC upkeep during indwell and lead to a new field of factors that should be analysed. This becomes of important concern when considering the yearly volume of PVC-carrying patients. The insertion sites of PVC inserted in routine in the hospital setting should be better monitored to prevent potential harms during patient care, including a minimum of 48h of post-removal surveillance. Further studies considering practice-mirroring factors in further detail, notably events of factor combinations or duration of the quality flaw are required.

## Supporting Information

S1 File Clearance from the French Advisory Committee for Data Processing in Health Research (CCTIRS). (PDF)

S2 File Clearance from the French Data Protection Authority (CNIL). (PDF)

S3 File Adult Information letter. (PDF)

S4 File Minor Child's Information letter. (PDF)

S5 File Parent Information letter. (PDF)

S1 Table Kaplan-Meier estimates of time to first PVC-related adverse event occurrence. (XLSX)

S2 Table Kaplan-Meier estimates of time to first PVC-related adverse event occurrence per event class. (XLSX)
