# Supplementary material for: Peripheral Venous Catheter-Related Adverse Events: Evaluation from a Multicentre Epidemiological Study in France (the CATHEVAL Project)
Source: PLoS One. 2017 Jan 3;12(1):e0168637. doi: 10.1371/journal.pone.0168637 (PMC5207628; doi:10.1371/journal.pone.0168637)
Supplement: S1 File — (PDF) [file pone.0168637.s001.pdf]

Liberté • Égalité • Fraternité
RÉPUBLIQUE FRANÇAISE

MINISTÈRE
DE L'ENSEIGNEMENT SUPÉRIEUR
ET DE LA RECHERCHE

**DIRECTION GENERALE POUR LA RECHERCHE
ET L'INNOVATION**

**Comité consultatif sur le traitement de l'information
en matière de recherche dans le domaine de la santé**

Dossier n° 13.719

Intitulé de la demande : ETUDE CATHEVAL. Evaluation des pratiques et analyse des risques associés aux cathéters veineux périphériques en milieu hospitalier : une étude multicentrique observationnelle.

Responsable scientifique : Pascal ASTAGNEAU
Cclin Paris-Nord- Site Broussais
96 rue Didot
75014 PARIS

Demandeur : **Christophe MISSE**
Hôpital Saint-Louis - APHP
Département de la Recherche Clinique et du Développement
Carré Historique – Secteur gris – Porte 23
1, avenue Claude Vellefaux
75475 PARIS Cedex 10

Dossier reçu le : 26.09.13

Dossier examiné le : **21 novembre 2013**

Avis du Comité consultatif :

Avis favorable

Toutefois,
. pour l'identification du CRF : il est préférable que le cahier d'observation soit nominatif tant qu'il est dans l'établissement mais anonymisé quand les informations sortent de l'établissement. La mention des 3 initiales du nom ne paraît donc pas utile. L'étiquette patient, nécessaire pour la gestion du recueil de données, doit être retirée avant l'envoi des documents au CCLIN (la date de naissance peut être remplacée par l'âge) ; les différentes fiches ne comporteront alors que le numéro d'anonymisation de l'étude. La liste de correspondance (identité exacte / numéro de l'étude) qui sera conservée dans chaque centre permettra de retrouver le dossier du patient si besoin. La partie identifiante du CRF doit figurer sur une page à part facilement détachable,
. la liste de correspondance peut être détruite après le gel de la base, ceci ne permettra plus d'accéder aux données (dans ce cas le mentionner dans la note d'information) mais garantira l'anonymat,
. dans la note d'information : retirer « mademoiselle » de la note adulte. Prévoir une note d'information adaptée pour les adolescents, qui peuvent s'opposer pour eux-mêmes à leur participation, même si les titulaires de l'autorité parentale ne s'y opposent pas. Dans la note pour les parents, remplacer « vos données médicales » par « les données médicales de votre enfant »,
. les participants peuvent être tenus informés des résultats globaux de l'étude à leur demande : le mentionner dans la note d'information,
. se limiter à 1+1 initiales.

Fait à Paris, le 27novembre 2013

Jean-Louis Serre
Président du Comité consultatif
