# Supplementary material for: Peripheral Venous Catheter-Related Adverse Events: Evaluation from a Multicentre Epidemiological Study in France (the CATHEVAL Project)
Source: PLoS One. 2017 Jan 3;12(1):e0168637. doi: 10.1371/journal.pone.0168637 (PMC5207628; doi:10.1371/journal.pone.0168637)
Supplement: S2 File — (PDF) [file pone.0168637.s002.pdf]

CNIL

Le Vice-Président délégué

Monsieur Christophe MISSE
DIRECTEUR
ASSISTANCE PUBLIQUE - HOPITAUX DE PARIS - HOPITAL BICHAT - CLAUDE BERNARD
AP-HP / DRCD
46 RUE HENRI HUCHARD
75018 - PARIS

Paris, le

16 OCT. 2014

N/Réf. : MMS/VCS/AR1410374

Objet : NOTIFICATION D'AUTORISATION

Décision DR-2014-463 autorisant l'ASSISTANCE PUBLIQUE - HOPITAUX DE PARIS - HOPITAL BICHAT - CLAUDE BERNARD à mettre en œuvre un traitement de données ayant pour finalité une étude sur l'évaluation des pratiques et analyse des risques associés au cathéter veineux périphériques en milieu hospitalier. (Demande d'autorisation n° 914068)

Monsieur le Directeur,

Vous avez saisi notre Commission d'une demande d'autorisation relative à un traitement de données à caractère personnel ayant pour finalité :

ÉTUDE SUR L'ÉVALUATION DES PRATIQUES ET ANALYSE DES RISQUES ASSOCIÉS AU CATHETER VEINEUX PÉRIPHERIQUES EN MILIEU HOSPITALIER.

Cette étude, bénéficiant d'un avis favorable du CCTIRS a pour objectif de déterminer les moyens de prévention institutionnels, organisationnels, environnementaux, individuels et d'équipe à mettre en œuvre pour limiter les complications sur cathéters veineux périphériques. Elle sera menée sur une population de patients adultes de plus de 15 ans, hospitalisés pour plus de 24heures dans les services de médecine et chirurgie de 8 centres investigateurs en France et porteurs d'un cathéter veineux périphérique.

Ce traitement relève de la procédure des articles 54 et suivants de la loi du 6 janvier 1978 modifiée.

Les services de notre Commission ont étudié les conditions définies dans le dossier de formalités préalables déposé à l'appui de cette demande et notamment celles relatives à l'exercice effectif des droits des participants à l'étude.

Après avoir examiné les catégories de données traitées et les destinataires, je vous rappelle que conformément au 3ème alinéa de l'article 55, la présentation des résultats du traitement de données ne peut, en aucun cas, permettre l'identification directe ou indirecte des personnes concernées.

Les données nécessaires au traitement des courriers et des dossiers de formalités reçus par la CNIL sont enregistrées dans un fichier informatisé réservé à son usage exclusif pour l'accomplissement de ses missions. Vous pouvez exercer votre droit d'accès aux données vous concernant et les faire rectifier en vous adressant au correspondant informatique et libertés (CIL) de la CNIL.

En application des articles 15 et 69 de la loi précitée et de la délibération n° 2014-073 du 4 février 2014 portant délégation de pouvoirs de la Commission nationale de l'informatique et des libertés à son président et à son vice-président délégué, j'autorise la mise en œuvre de ce traitement.

Je vous prie d'agréer, Monsieur le Directeur, l'expression de mes salutations distinguées.

Marie-France MAZARS
