# Supplementary material for: Peripheral Venous Catheter-Related Adverse Events: Evaluation from a Multicentre Epidemiological Study in France (the CATHEVAL Project)
Source: PLoS One. 2017 Jan 3;12(1):e0168637. doi: 10.1371/journal.pone.0168637 (PMC5207628; doi:10.1371/journal.pone.0168637)
Supplement: S5 File — (PDF) [file pone.0168637.s005.pdf]

ETUDE CATHEVAL
Evaluation des pratiques et analyse des risques associés aux cathéters veineux périphériques en milieu hospitalier : une étude multicentrique observationnelle

Cette recherche est organisée par le CClin Paris-Nord
(Centre de coordination de la lutte contre les infections nosocomiales de l'inter-région Nord)
et encadrée par le Département de la Recherche Clinique et du Développement
de l'Assistance Publique - Hôpitaux de Paris

NOTE D'INFORMATION Note pour les titulaires de l'autorité parentale sur un enfant participant à la recherche biomédicale :

Madame, Monsieur,

Nous proposons que votre enfant participe à un projet de recherche clinique mené actuellement dans le service où votre enfants est hospitalisé(e) : elle concerne les cathéters utilisés pour les perfusions (cathéters veineux périphériques). Soucieux de la qualité des soins et de la sécurité des patients, votre service a choisi de participer à cette étude pour aider à l'amélioration des pratiques et de l'organisation des soins liés au cathéter.
Merci de lire attentivement cette note d'information qui vous explique les objectifs et les modalités de cette étude. Si besoin, n'hésitez pas à demander des explications.

Quel est le but de la recherche ?

Cette recherche porte sur les complications (sans gravité dans la majorité des cas) qui peuvent parfois survenir lors de l'utilisation de ces cathéters. Le but est de comprendre comment ces complications peuvent survenir.
Pour cela, il faut inclure dans l'étude environ 400 patients porteurs de cathéters, âgés de plus de 15 ans, dans différents services. Une dizaine d'établissements situés en Ile de France et en Haute-Normandie sont actuellement volontaires pour participer à cette étude.

En quoi consiste la recherche ?

Dans le cadre de la prise en charge habituelle de la pathologie de votre enfant, un cathéter veineux périphérique habituellement utilisés dans le service est posé à votre enfant pour une perfusion, le personnel soignant lui prodiguera les soins habituels (entretien et surveillance du cathéter). Pour les besoins de l'étude, et si vous êtes d'accord, nous aurons besoin de collecter quelques données concernant toute intervention liée au cathéter posé à votre enfant .
Pour ce faire, une personne de l'équipe d'hygiène de l'établissement viendra observer comment le cathéter est posé (si le cathéter est posé un lundi). Une autre personne du service où est hospitalisé votre enfant vérifiera et notera tous les jours si tout se passe bien au niveau du cathéter de votre enfant.
Ces observations se dérouleront pendant toute la durée du maintien et 48h après le retrait du catheter.

Le recueil de ces données durera dans le service pendant 4 à 8 semaines.

Que vont devenir les données recueillies pour la recherche ?

Toutes les informations seront notées dans des fiches de recueil. Les données de votre enfant seront identifiées au cours de l'étude grâce aux 3 premières lettres de son nom et remplacées par un code à plusieurs chiffres (numéro d'anonymat) lors de la saisie informatique des données. A la fin de l'étude, les données recueillies dans le service pour l'ensemble des patients inclus seront analysées sous la responsabilité du professeur Astagneau Investigateur coordonnateur de l'étude (Centre de coordination de la lutte contre les infections nosocomiales de l'inter-région Nord).
En cas d'observation de complications, le service se réunira pour analyser les circonstances de survenue et réfléchir aux améliorations nécessaires.

Comment cette recherche est-elle encadrée ?

Cette étude, coordonnée par le CClin, est encadrée par le Département de la Recherche Clinique et du Développement de l'Assistance Publique - Hôpitaux de Paris et soutenue par le Ministère de la Santé. Elle a reçu l'avis favorable du Comité consultatif sur le traitement de l'information en matière de recherche dans le domaine de la santé (CCTIRS) et de la Commission nationale informatique et liberté (CNIL).

Quels sont vos droits ?

La participation de votre enfant à cette étude est entièrement libre et volontaire. Votre choix n'entraînera aucun préjudice sur la qualité des soins et des traitements qu'il est en droit d'attendre et sans conséquence sur la relation avec l'équipe paramédicale et médicale qui le prend en charge habituellement.
Vous pourrez tout au long de l'étude demander des explications sur le déroulement de l'étude aux personnes qui le suivent.
Conformément aux dispositions de la CNIL (loi relative à l'informatique, aux fichiers et aux libertés), vous disposez d'un droit d'accès et de rectification. Vous disposez également d'un droit d'opposition à la transmission des données couvertes par le secret professionnel susceptibles d'être utilisées et traitées dans le cadre de cette étude. Ces droits s'exercent auprès du l'équipe soignante en charge de la recherche qui seule connaît l'identité de votre enfant.

Si vous le souhaitez, à son terme, vous pourrez être informé par le médecin des résultats globaux de cette recherche.

Vous pouvez également accéder directement ou par l'intermédiaire d'un médecin de votre choix à l'ensemble des données médicales de votre enfant en application des dispositions de l'article L 1111-7 du Code de la Santé Publique.

Vous pouvez vous opposer à cette étude sans que cela ait d'influence sur la prise en charge de votre enfant. Si tel est le cas, nous vous demandons de le signaler au médecin qui le prend en charge.
